# Transcriptome analysis of *Brachypodium* during fungal pathogen infection reveals both shared and distinct defense responses with wheat

**DOI:** 10.1038/s41598-017-17454-3

**Published:** 2017-12-08

**Authors:** Jonathan J. Powell, Jason Carere, Gaurav Sablok, Timothy L. Fitzgerald, Jiri Stiller, Michelle L. Colgrave, Donald M. Gardiner, John M. Manners, John P. Vogel, Robert J. Henry, Kemal Kazan

**Affiliations:** 1 Commonwealth Scientific and Industrial Research Organization Agriculture and Food, St Lucia, Queensland, 4067 Australia; 2 Queensland Alliance for Agriculture and Food Innovation (QAAFI), University of Queensland, St Lucia, 4067 Queensland, Australia; 3 Plant Functional Biology and Climate Change Cluster (C3), University of Technology Sydney, PO Box 123, Broadway, NSW 2007 Sydney Australia; 4 Commonwealth Scientific and Industrial Research Organization Agriculture and Food, Black Mountain, Australian Capital Territory 2601 Australia; 5 Joint Genome Institute, United States Department of Energy, Walnut Creek, CA 94598 USA

## Abstract

Fusarium crown rot (FCR) of wheat and barley, predominantly caused by the fungal pathogen *Fusarium pseudograminearum*, is a disease of economic significance. The quantitative nature of FCR resistance within cultivated wheat germplasm has significantly limited breeding efforts to enhanced FCR resistance in wheat. In this study, we characterized the molecular responses of *Brachypodium distachyon* (*Brachypodium* hereafter) to *F*. *pseudograminearum* infection using RNA-seq to determine whether *Brachypodium* can be exploited as a model system towards better understanding of *F*. *pseudograminearum*-wheat interaction. The transcriptional response to infection in *Brachypodium* was strikingly similar to that previously reported in wheat, both in shared expression patterns of wheat homologs of *Brachypodium* genes and functional overlap revealed through comparative gene ontology analysis in both species. Metabolites produced by various biosynthetic pathways induced in both wheat and *Brachypodium* were quantified, revealing a high degree of overlap between these two species in metabolic response to infection but also showed *Brachypodium* does not produce certain defence-related metabolites found in wheat. Functional analyses of candidate genes identified in this study will improve our understanding of resistance mechanisms and may lead to the development of new strategies to protect cereal crops from pathogen infection.

## Introduction

Cereal infecting pathogens belonging to the genus *Fusarium* cause several diseases of economic significance including Fusarium head blight, Fusarium crown rot and Fusarium root rot^1–3^. The development of resistant germplasm is a preferred strategy to manage these pathogens. However, identifying and characterizing sources of resistance to these pathogens has proven difficult due to the highly quantitative basis of resistance and the complex nature of the wheat genome. Global transcriptomic analyses of the host response to Fusarium crown rot (FCR) in wheat using microarray technology have previously identified infection-inducible genes in different wheat cultivars. Genes encoding anti-microbial peptides, metabolic enzymes and regulatory proteins such as protein kinases and transcription factors in wheat have been implicated in plant defence and disease resistance^4^. However, subsequent functional analyses of defense-associated genes through mutational approaches have been hampered due to the highly complex and polyploid nature of the wheat genome^5^. Analyzing the transcriptional response to FCR in wheat is further complicated due to a phenomenon called “homoeolog expression bias” which causes differential expression patterns between homoeologs for various defense related pathways particularly from B and D subgenomes globally^6^. Dissecting the molecular responses to FCR in a model species may, therefore, be beneficial in determining how effective resistance against this pathogen can be achieved in wheat^7^.

*Brachypodium distachyon* (*Brachypodium* hereafter) is a monocot model with advantages such as short generation time, small and diploid genome and extensive genetic and genomic resources^8–10^ (e.g. fully sequenced genome and mutant resources) and amenability to transformation^11,12^. It is estimated that *Brachypodium* and wheat diverged from a common ancestor relatively recently^13^. Therefore, *Brachypodium* shares highly conserved gene synteny and close sequence homology with hexaploid wheat^13^.

As a first step towards developing *Brachypodium* as a model for cereal-pathogen interactions, previous work has focused predominantly on testing the infectibility of *Brachypodium* by major cereal pathogens such as *Puccinia* spp., *Fusarium graminearum*, *Fusarium culmorum*, *Ramularia collo-cygni*, *Oculimacula* spp., *Rhizoctonia solani* and *Claviceps purpurea* ^14–18^. *Brachypodium* can also be infected by *F*. *pseudograminearum* ^19^, suggesting that this model can be used for characterizing the molecular basis of resistance to this pathogen in wheat and barley. Transcriptomic studies have also been performed to study responses to abiotic^20,21^ and biotic stress^22–24^ as well as phytohormones in *Brachypodium* ^25^. However, little work has been performed to compare molecular responses activated in wheat and *Brachypodium* during pathogen infection, though such approaches have been aided by the release of genome assemblies and annotations for both wheat^26^ and *Brachypodium* ^13^.

Although *Brachypodium* is proposed to be a good model for cereal-pathogen interactions, to the best of our knowledge, there has not been a systematic comparison of defense responses triggered by pathogen attack in wheat and *Brachypodium* under the same experimental conditions. In this study, to provide insights into defense-associated processes, we first identified *Brachypodium* genes responding to fungal infection using high throughput RNA-seq profiling. Secondly, we compared the defense-associated transcriptome of wheat and *Brachypodium* during infection by *F*. *pseudograminearum*, followed by targeted metabolomics analyses in both species^27^. The results show that while molecular responses of these two species to *F*. *pseudograminearum* are largely conserved at the transcriptome level, certain metabolic differences are also notable. In particular, we found that certain indole-derived defense compounds are produced only in wheat but not in *Brachypodium*, despite the induction of corresponding genes in the production of these metabolites in both species. Overall, our results provide new insights into *Brachypodium-*pathogen interaction and validates the overall suitability of *Brachypodium* as a model for the Fusarium crown rot interaction in wheat despite some differences between the crop and model. Future work will exploit the expanding genetic resources within *Brachypodium* to discover novel mechanisms of resistance with a view to improving necrotrophic pathogen resistance in wheat.

## Results and Discussion

### Assessing the transcriptomic response of *Brachypodium* to *Fusarium* infection

RNA-seq analysis was performed on the *Brachypodium* community standard line (Bd21-3) since this line has a suitable genome reference and annotation available^13^ for reference-based transcriptome analysis and a large collection of publically available mutant lines has been generated in this background^8,9^ to facilitate future functional characterization of genes identified within this study. Preliminary pathology assays indicated Bd21-3 was relatively resistant compared to other *Brachypodium* natural accessions and developed similar symptoms and disease progression to that observed in hexaploid wheat. In total, 2498 genes were differentially expressed (DE) in *Brachypodium* during *Fusarium* infection at 3 dpi. In total, 1448 genes were up-regulated under infection with fold-changes from 240 to 1.17 (Fig. S1; Table S1) while 1050 genes were down-regulated after infection with a minimum fold-change of 0.85 (expressed 1.17-fold higher in mock compared to infected) and a maximum fold-change of 0.24 (expressed 4.16-fold higher in mock compared to infected) (Table S1).

In order to confirm DE of genes of interest, an independent infection time-course was performed, incorporating 1, 3 and 7 dpi time-points. The genes to be validated were selected based on their similar magnitude of fold-change in both *Brachypodium* (this study) and wheat^27^ and their functional categories such as defense, signaling and transport as well as primary and secondary metabolism. Fourteen genes selected for validation were found to be similarly up- or down-regulated in the validation time-course at one or more time-points (Fig. S2), demonstrating the robustness of the RNA-seq data.

### Enriched molecular functions and processes within the response to infection

To determine if the DE genes were enriched for particular biological functions we examined their Gene Ontology (GO) and InterPro classification using Singular Enrichment Analysis (SEA) and Fisher’s exact test with Bonferroni correction for multiple comparisons with *p*-value cutoff < 0.01 (Fig. S3; Table S2). These analyses revealed enrichment of protein phosphorylation GOs in up-regulated gene sets and enrichment of UDP-glucuronosyl/UDP-glucosyltransferase [IPR002213], glutathione S-transferase, N-terminal [IPR004045], glutathione S-transferase, C-terminal-like [IPR010987], WRKY domain [IPR003657], sugar/inositol transporter [IPR003663] and ABC transporter-like [IPR003439] as enriched protein domains. The abundance of the UDP-glucuronosyl/UDP-glucosyltransferase [IPR002213] in the up-regulated gene sets correlates with the role of UDP-glucosyltransferases in the detoxification of the toxin deoxynivalenol (DON)^28^. Notably, the enrichment of the glutathione S-transferase in up-regulated genes is further supported by KEGG Orthology Based Annotation System (KOBAS) analysis which identifies enrichment in Kyoto Encyclopedia of Genes and Genomes (KEGG) pathways, revealing statistical enrichment of the glutathione metabolism pathway (bdi00480) (p-value = 1.1 × 10^−08^; FDR = 5.9 × 10^−07^).

We also observed enrichment of protein domains such as tubulin/FtsZ, C-terminal [IPR008280], tubulin/FtsZ, 2-layer sandwich domain [IPR018316], tubulin/FtsZ, GTPase domain [IPR003008], nucleotide-diphospho-sugar transferases [IPR029044] and all forms of tubulin [IPR000217] domains among the most abundant categories for down-regulated genes. Notably, the enrichment of the GTPase domain and tubulin/FtsZ suggests down-regulation of chloroplast binary fission and division possibly indicating loss of photosynthetic efficiency during pathogen infection^29,30^.

Alternative splicing plays a global role in regulating the protein diversity and has been widely linked to biotic and abiotic stresses in model plants, including *B*. *distachyon* ^31–33^. Since intron retention events have been shown to be the predominant form of alternative splicing in plants^32^, we specifically looked for the intronic splice signal occurring during pathogen infection by performing a genome-wide mapping of the mock and *Fusarium* treated reads and by applying a count based Bayesian model as implemented in rMATS^34^ with a cut off splicing difference of 0.001. This analysis identified a total of the 132 intron retention events based on significant junctions (adjusted *p* < 0.01) and a total of 139 intron retention events (Table S3) based on significant junctions and reads on targets (adjusted *p* < 0.01). Previous studies in barley detail the role of the powdery mildew-induced mRNAs as alternatively spliced with the powdery mildew resistance gene *Rar1* producing two transcripts with retained intron^35^. We found differentially-spliced auxin response factors (*ARF*s) in the category of the retained introns. *ARF*s are known to affect resistance to *Fusarium* pathogens^36,37^.

Differentially expressed genes revealed as a result of the expression analysis belong to a number of other categories as briefly described below:

#### PR Genes

Genes encoding pathogenesis related proteins including two *PR1* homologs, four beta-glucanases (*PR2*), three chitinases (*PR3*), two *PR5-like* genes and one *PR10* were differentially expressed (Table S1). Quantitative Real Time Polymerase Chain Reaction (qRT-PCR) was used to quantify expression of selected pathogen-related genes in a separate experiment. *PR1*.*1* was up-regulated approximately 70 fold at 7 dpi while *PR2*-*like* gene was up-regulated ~100 fold. We also independently confirm that two *PR3-like* (chitinase) genes (Bradi3g48230 and Bradi3g32340) (~100 fold and ~40 fold) and one peroxidase (Bradi1g17840) (~10 fold) were highly induced at 7 dpi (Table S1).

#### Genes involved in reactive oxygen species metabolism

Similar to responses observed in wheat^27^, infection of *Brachypodium* with Fusarium has led to increased expression of genes encoding enzymes involved in the production of reactive oxygen species (ROS). We identified five DE genes encoding oxalate oxidases and germin-like proteins up-regulated within infected plants (Table S1). Previous studies have widely elucidated the oxidative burst of H_2_O_2_ post-Fusarium infection in wheat^38,39^. Using the differentially expressed genes and KOBAS analysis, we identified the glutathione-S-transferases (GST) pathway as statistically enriched pathway (Table 1), indicating the role of GSTs being up-regulated most likely to combat the effects of excessive H_2_O_2_ production during the infection process.

**Table 1:** Differentially expressed UDP-glycosyltransferase, tryptophan biosynthesis and metabolism and glutathione-S-transferase genes by *F*. *pseudograminearum* at 3 dpi (expressed as fold-change) in *Brachypodium* (this study) and their homologs in wheat subgenomes as reported previously^27^. Infinite indicates expression was only detected within infected samples.

BradiID	Gene Description	Fold-Change (FC)	*p*-adj	A subgenome homolog FC	B subgenome homolog FC	D subgenome homolog FC
**UDP-glycosyltransferases**						
Bradi5g03310	udp-glycosyltransferase 74f2-like	Infinite	0.0018			
Bradi5g03300	indole-3-acetate beta-glucosyltransferase	11.580	5.00e^−05^			
Bradi5g03380	udp-glycosyltransferase 74f2-like	6.792	5.00e^−05^	—	7.287	7.006
Bradi1g45950	udp-glycosyltransferase 73c5-like	4.374	5.00e^−05^			
Bradi5g16100	udp-glycosyltransferase 73d1-like	4.070	5.00e^−05^	1.958	1.720	—
Bradi3g46867	udp-glycosyltransferase 85a2-like	2.459	5.00e^−05^			
Bradi3g27621	udp-glycosyltransferase 1	2.329	0.00145			
Bradi1g08190	udp-glycosyltransferase 83a1-like	2.247	5.00e^−05^			
**Tryptophan biosynthesis and metabolism**						
Bradi1g76800	anthranilate chloroplastic-like	2.184	5.00e^–05^			
Bradi4g08830	indole-3-glycerol phosphate chloroplastic-like	11.415	5.00e^–05^			
Bradi5g05430	indole-3-glycerol phosphate synthase	3.307	5.00e^–05^	—	2.005	—
Bradi1g55440	tryptophan synthase alpha chain-like	3.762	5.00e^–05^			
Bradi1g35600	tryptophan synthase beta chain	4.702	5.00e^–05^	—	1.358	—
Bradi3g14490	tryptophane synthase1	2.829	5.00e^–05^			
Bradi3g14760	aromatic-l-amino-acid decarboxylase	11.774	0.00095	41.812	—	—
Bradi3g14740	aromatic-l-amino-acid decarboxylase	6.973	0.00045	—	Inf	—
Bradi4g39240	cytochrome p450 71a1-like	4.431	5.00e^–05^			
**Glutithione-S-transferases**						
Bradi3g31720	probable glutathione s-transferase gstu6-like	56.978	0.0037			
Bradi1g34727	probable glutathione s-transferase gstu6-like	6.766	5.00e^–05^			
Bradi3g31727	probable glutathione s-transferase gstu6-like	5.893	5.00e^–05^	7.299	3.673	1.924
Bradi3g10950	glutathione s-transferase omega-like 2-like	5.742	5.00e^–05^	4.765	7.818	—
Bradi3g31737	probable glutathione s-transferase gstu6-like	5.724	5.00e^–05^			
Bradi2g13120	glutathione transferase	5.240	5.00e^–05^	—	3.589	—
Bradi2g42260	probable glutathione s-transferase gstu6-like	4.609	5.00e^–05^			
Bradi3g31850	probable glutathione s-transferase gstu6-like	3.674	5.00e^–05^			
Bradi3g31880	probable glutathione s-transferase gstu6-like	3.431	5.00e^–05^	3.003	7.457	—
Bradi3g31777	probable glutathione s-transferase gstu6-like	3.351	5.00e^–05^			
Bradi2g13010	glutathione s-transferase 4-like	2.417	0.00015			
Bradi2g13020	glutathione s-transferase 4-like	2.289	5.00e^–05^			
Bradi2g55892	glutathione transferase gst 23-like	2.234	5.00e^–05^	—	1.628	—

#### Genes associated with pathogen sensing and signaling

Multiple genes putatively involved in pathogen reception and signaling such as leucine rich repeat receptor-like kinases (RLKs), cysteine rich receptor-like kinase and lectin-domain containing receptor kinases were identified within the dataset. The role of LRK receptors has been well characterized in other pathogen interactions, in particular those involving biotrophic pathogens^40^. However, relatively little work has been performed on their roles in perceiving signals from necrotrophic fungal pathogens. Six cysteine-rich receptor-like kinases, four leucine-rich repeat and ten lectin-rich repeat kinase-encoding genes were differentially expressed at 3 dpi in response to infection in *Brachypodium* (Table S1). Several genes annotated as disease resistance (R) proteins were also significantly induced (Table S1). For instance, Bradi2g59310 annotated as disease resistance protein RPP13-like was the most highly induced gene with ~200 fold up-regulation. Other resistance-related genes differentially expressed included Bradi2g43120 (~17 fold), Bradi1g70730 (~3.8 fold) Bradi2g18851 (~2.6 fold) annotated as pleiotropic drug resistance protein 3-like, disease resistance response and disease resistance protein RGA3-like respectively and also Bradi2g01320, a homolog of the leaf rust resistance gene *Lr10* (~2.7 fold).

#### Genes encoding transcription factors

Identifying transcription factors (TFs) induced during infection provides targets for functional characterization approaches to understand how perception of the pathogen leads to local and systemic signaling. Indeed, TFs were well represented within the dataset and in many cases showed high induction by *F*. *pseudograminearum*. Classes of TFs highly represented include WRKY, MYB, NAC and basic helix loop helix (bHLH) TFs (Table S1). TFs validated by qRT-PCR revealed one MYB TF induced both at 3 dpi (~3.5 fold) and at 7 dpi (~20 fold) and a WRKY TF at 3 dpi (~25 fold) and at 7 dpi (~225 fold). Two NAC TFs down-regulated by *F*. *pseudograminearum* in the RNA-seq data (Bradi5g12407 – 0.62 fold-change) were also confirmed to be down-regulated at 3 dpi and 7 dpi to approximately 0.3 fold in both cases (Table S1).

#### Genes encoding transporters

Transporter proteins were also highly represented within the dataset having 133 DE genes associated with the ‘Transport’ GO term specifically trans-membrane transport, monosaccharide transport and organic hydroxy compound transport terms (Supplementary File 2). Eight ATP-binding cassette (ABC) transporters were induced more than 2 fold (Table S1). The two ABC transporters observed were also up-regulated at later time points with Bradi2g10110 induced 17 fold (3 dpi) and 220 fold (7 dpi) and Bradi3g34890 induced 3.5 fold (3 dpi) and 7.5 fold (7 dpi) (Supp. File 3). ABC transporters play important roles during plant defense. For instance, one ABC transporter encoded by *Lr34* has been shown to provide durable adult plant resistance to multiple rust diseases^41^. Five wheat ABC transporters have been previously shown to be induced during infection by *F*. *graminearum* as well after exogenous application of the *Fusarium* toxin deoxynivalenol (DON)^42^. Therefore, the induction of these genes encoding putative transporters in *Brachypodium* seems to be consistent with the response observed in wheat, resulting in the activation of a strong defense response.

#### Comparison of differential gene expression during *Fusarium* infection in wheat and *Brachypodium*

**Figure 1:**
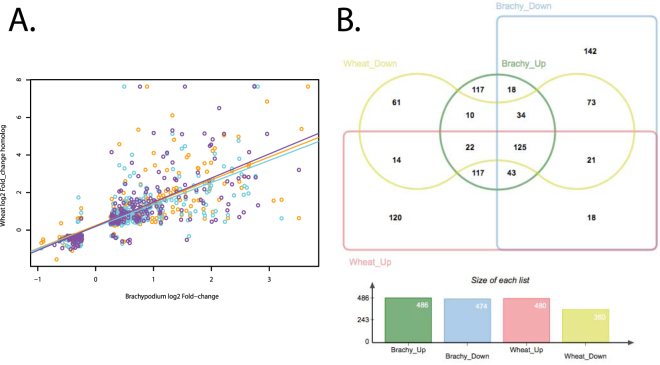
Panel A: Comparison of differentially expressed gene fold-changes between *Brachypodium* genes and their wheat homologs also differentially expressed during *Fusarium* infection. Datapoints represent comparison between a *Brachypodium* gene and its reciprocal best BLAST A subgenome homolog (orange), B subgenome homolog (cyan) and D subgenome homolog (purple). Panel B: Venn diagram showing the functional convergence and dis-convergence between *Brachypodium* and wheat during infection.

To determine whether *Brachypodium* and wheat respond to infection by *F*. *pseudograminearum* similarly, we compared the *Brachypodium* transcriptome data generated in this study with that for wheat^27^. The wheat transcriptome data used here were generated using similar age (6 days post germination) wheat seedlings exposed to pathogen infection side by side with the *Brachypodium* seedlings reported here and inoculated using the same inoculum to minimize potential differences due to any environmental factors in this comparison^27^. In order to appropriately assess the similarity of response to *F*. *pseudograminearum* infection in wheat and *Brachypodium*, a bioinformatic analysis was developed to identify wheat homologs of *Brachypodium* genes within each wheat subgenome with reciprocal best BLAST (RBB) between A, B and D sub-genomes independently (A vs. B, B vs. D, A vs. D) and identify RBB homologs between *Brachypodium* and each wheat sub-genome. Global RBB searches of *Brachypodium* genes to the wheat chromosomal survey sequence showed that approximately 30% of *Brachypodium* genes were able to find a homolog match in at least one wheat sub-genome. In total, 501 differentially expressed *Brachypodium* genes (~20% total DE genes) had a RBB homolog from one or more wheat sub-genomes also differentially expressed with 239 from the A genome (76 unique), 283 from the B genome (129 unique) and 256 from the D genome (93 unique). Seventy-four differentially expressed *Brachypodium* genes had RBB homologs from all three sub-genomes. Comparison of *Brachypodium* gene expression values to those in wheat in a sub-genome specific manner revealed a highly correlated pattern of expression for homologous genes between the two species (Fig. 1A). Analysis of correlation (Spearman ranking) revealed highly significant correlation between log2 fold-change values of DE genes in *Brachypodium* and identified homologs across the whole wheat genome (*r = *0.83), A genome RBB homologs (*r = *0.85), B genome RBB homologs (*r = *0.81) and D genome RBB homologs (*r* = 0.82) respectively.

In addition, comparison of global differential expression by biological processes and molecular functions undergoing change as inferred by GO term enrichment highlights the similarity in molecular response observed in *Brachypodium* and wheat. For the identification of conserved functional roles across *Brachypodium* and wheat during infection, up- and down-regulated genes from *Brachypodium* and wheat were analyzed for the associated GO term. GO analysis assigned a total of 486 and 474 ontology terms in *Brachypodium* up- and down-regulated genes and a total of 480 and 360 GO terms for wheat (Fig. 1B). Venn analysis revealed a total of 117 and 73 ontology terms shared between *Brachypodium* and wheat up- and down-regulated genes. Additionally, a total of 114 and 172 GO terms were found specifically in *Brachypodium* up- and down regulated genes.

#### Genes putatively involved in detoxification of deoxynivalenol

Plants have evolved mechanisms to detoxify compounds produced by pathogens by adding sugar moieties or other functional groups^43,44^ or through enzymatic degradation. UDP-glycosyltransferases able to detoxify DON by adding a glycosyl group to the compound have been identified in barley^45^ and *Brachypodium* ^46^. We identified eight UDP-glycosyltransferase encoding genes induced in *Brachypodium* during infection (Table 1). Several up-regulated UDP-glycosyltransferases (Bradi5g03300, Bradi5g03380 and Bradi5g03390) have also been previously shown to be highly induced during Fusarium head blight infection and after exogenous application of DON in *Brachypodium* ^46^. One enzyme encoded by Bradi5g03300 was shown to metabolize DON when expressed in yeast^46^. Three UDP-glycosyltransferases previously shown to be highly induced during Fusarium head blight and by DON treatment (Bradi5g03300, Bradi5g03380 and Bradi5g03390) were induced at 3 dpi (~350 fold, ~65 fold and ~6.2 fold) and then highly induced at 7 dpi (~2400 fold, ~830 fold and ~16 fold) (Supp. File 2). This indicated these genes may be responding to DON produced by the pathogen during infection and that DON detoxification is a defense strategy used by *Brachypodium* for combating the pathogen response.

#### DON is a virulence factor for *F*. *pseudograminearum* during infection of *Brachypodium*

**Figure 2:**
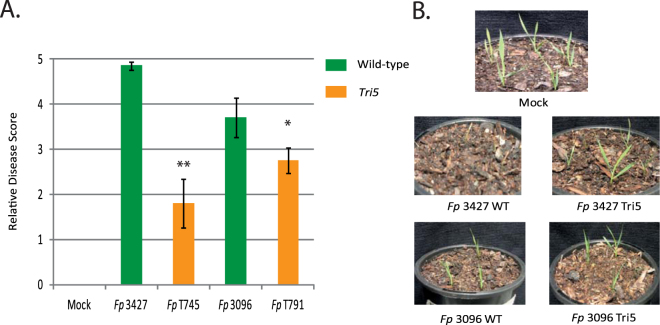
Reduction of virulence in *Tri5 F*. *pseudograminearum* knockout mutants. Panel A: Qualitative disease scores as a measure of isolate virulence. Plants inoculated with the wild-type parental strains showed significantly greater symptom development than the plants inoculated with the *Tri5* mutants. Student’s t-tests with statistically significant comparisons marked *p < 0.05 and **p < 0.01. Panel B: Photographs of representative pot-based infection assays with the parental isolates and *Tri5* mutants.

DON has been previously shown to be an important virulence factor for *F*. *graminearum* in establishing Fusarium head blight in wheat and *Brachypodium* ^23,47^. *F*. *pseudograminearum* has been shown to produce DON^48^ and to test the importance of DON for pathogen virulence during infection in *Brachypodium*, *F*. *pseudograminearum Tri5* knockout mutants^27^ were utilized in infection assays. Results from both pot-based inoculation and lab-based infection assays showed *Tri5* mutants had reduced virulence compared to wild-type strains (Fig. 2). Thus, it appears that the pathogen produces DON during infection and *Brachypodium* responds to this by producing UDP-glycosyltransferases known to detoxify DON by adding a glucose group, leading to high accumulation of DON-3-glucoside^49^. Together, these results show that similarly to its role during wheat-*F*. *pseudograminearum* interaction, DON is an important virulence factor during the colonization of *Brachypodium* by *F*. *pseudograminearum*.

#### Activation of the phenylalanine, tyrosine and tryptophan pathway in response to infection

**Figure 3:**
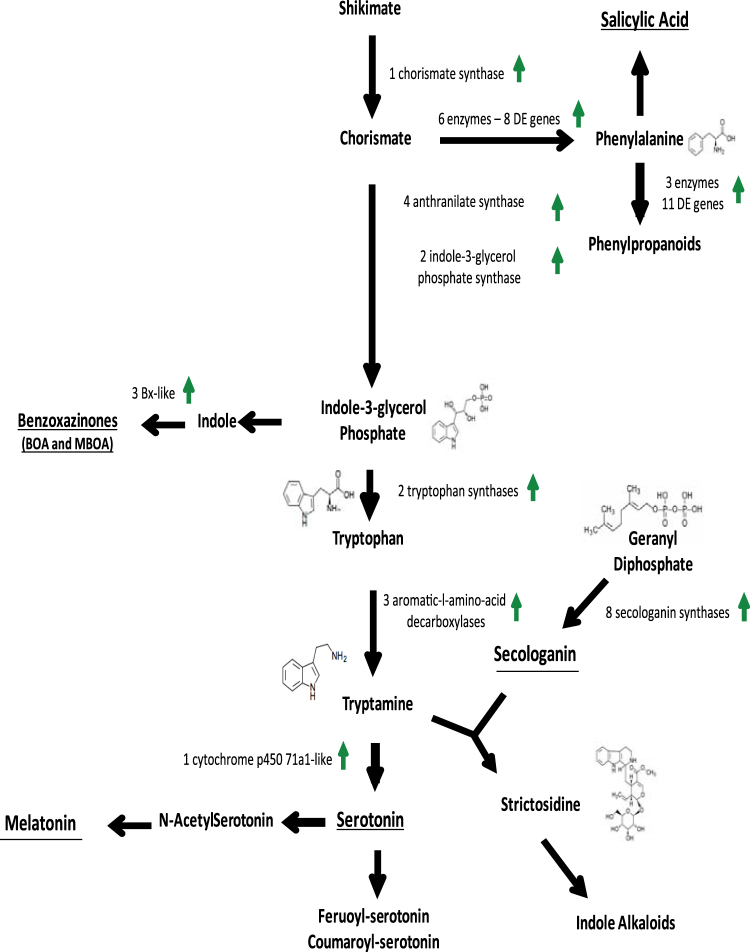
Metabolic pathway (modified from ref.^27^) displaying differentially expressed genes involved in phenylalanine, tryptamine and tyrosine metabolism. Green arrows denote up-regulated genes.

As was observed in wheat^27^ (Powell *et al*. 2017), increased activation of the pentose phosphate pathway in response to infection was evident with twelve up-regulated genes involved in production of D-Erythrose-4P. This metabolite is a key precursor within the phenylalanine, tyrosine and tryptophan (PTT) biosynthesis pathway; a pathway in which many DE genes function (Fig. 3). Phenylalanine production is important for defense against fungal pathogens in *Brachypodium*, demonstrated by increased susceptibility in RNAi mutants with attenuated expression of phenylalanine ammonia lyase genes against *F*. *culmorum* and *Magnaporthe grisea* ^50^. Phenylalanine also plays a role in defense as an important precursor for a multitude of metabolites including lignins^51,52^. Several peroxidases involved in lignin biosynthesis were also up-regulated in *Fusarium* infected plants (Table S1).

#### Induction of genes encoding plant hormones during *Fusarium* infection

Systemic and local signaling of pathogen response in plants relies on production of plant hormones (e.g. SA and jasmonic acid (JA)). Five 12-oxophytodinoate reductase (*OPR*) genes involved in JA biosynthesis were found to be significantly up-regulated at the 3 dpi time-point which provided molecular evidence for induction of the JA signaling pathway (Table 2). One *OPR3* encoding gene, Bradi2g35907, was found to be highly induced at 1 dpi (~3 fold), 3 dpi (~19 fold) and 7 dpi (~490 fold). In addition, several *ethylene response factors* (*ERF*s) were up-regulated. Genes typically involved in SA biosynthesis such as phenylalanine ammonia lyases and isochorismate synthases were not differentially expressed at 3 dpi though a single chorismate synthase (Bradi1g67790) was slightly induced (~1.2 fold).

**Table 2:** Differentially expressed 12-oxophytodienoate reductase genes by *F*. *pseudograminearum* at 3 dpi in *Brachypodium*.

BradiID	Gene Description	Fold-Change	*p*-adj
Bradi2g35907	12-oxophytodienoate reductase 1	24.919	5.00e^−05^
Bradi1g05870	12-oxophytodienoate reductase 1	4.624	5.00e^−05^
Bradi1g05880	12-oxophytodienoate reductase 1	3.472	5.00e^−05^
Bradi3g50490	ethylene-responsive transcription factor 1a-like	3.546	0.00005
Bradi2g11890	ethylene-responsive factor-like protein 1	2.526	0.00005

**Figure 4:**
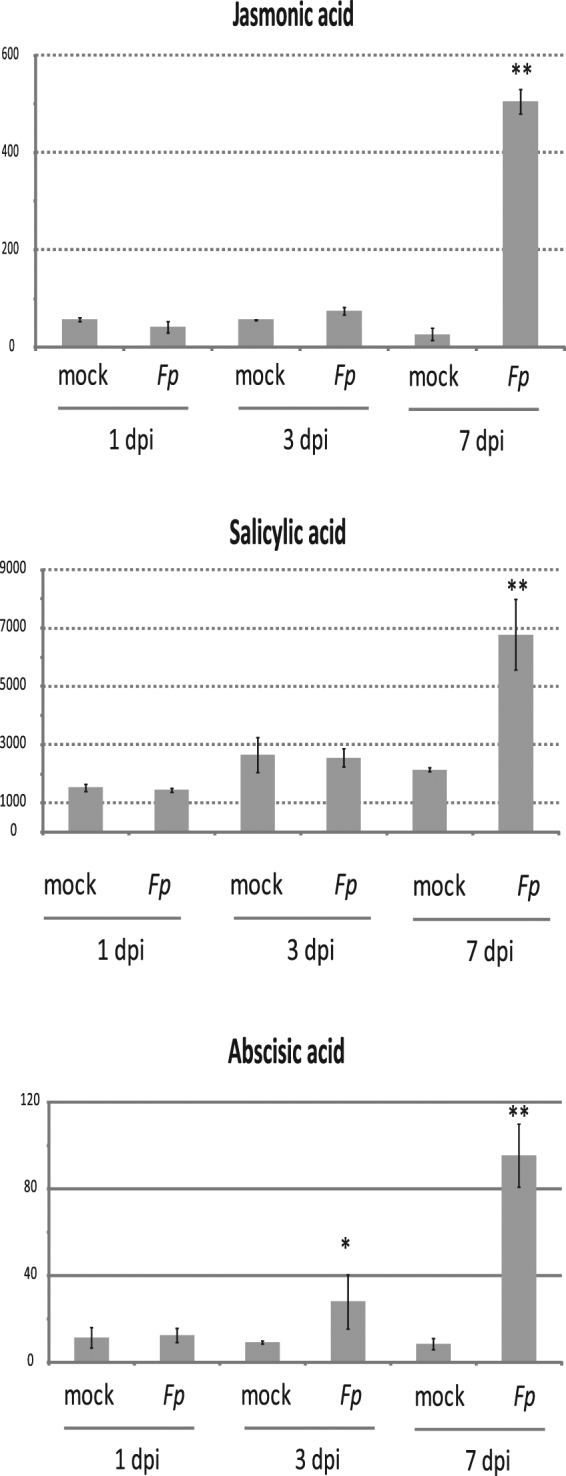
Induction of defense-related phytohormones during *F*. *pseudograminearum* infection in *Brachypodium*. Error bars display the standard error of the mean across three biological replicates. Student’s *t*-tests with statistically significant differences between mock and inoculated samples of the same time point were marked *p < 0.05 and **p < 0.01.

To determine whether observed changes in gene expression correlate with increased phytohormone levels, we measured SA, JA and ABA levels in infected plants. Results indicated detectable production of all hormones in *Brachypodium* at 1 dpi with no significant differences identified between mock- and infected-samples. Measurements of JA and ABA at 3 dpi revealed a statistically significant induction under pathogen treatment compared with mock while jasmonate-isoleucine (JA-Ile) and SA showed no significant difference (Fig. 4). SA and ABA both showed high induction as did both JA and JA-Ile under pathogen treatment at 7 dpi suggesting activation of both SA and JA-mediated signaling response during infection (Fig. 4). These observations are in line with the previously described roles of JA during *F*. *pseudograminearum*-wheat interaction^4,53,54^. However, it was interesting to observe high SA induction at the same timepoint since JA and SA signaling pathways act antagonistically in some plant species^55^. SA is known to play an important role in resistance to Fusarium head blight; posited to act through direct anti-fungal effect and activation of SA mediated defense pathways^56,57^.

### Differential accumulation of tryptophan-derived metabolites under pathogen infection in *Brachypodium* and wheat

**Figure 5:**
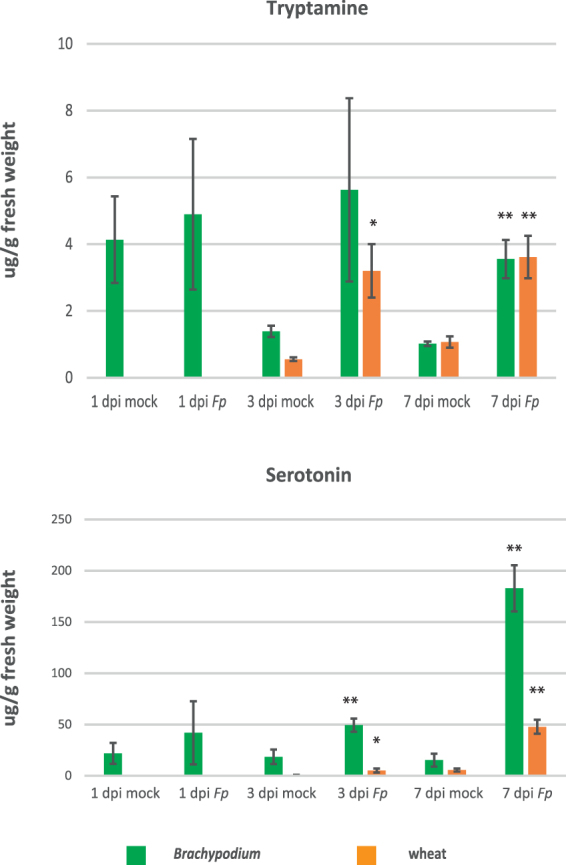
Induction of tryptamine and serotonin during *F*. *pseudograminearum* infection in *Brachypodium*. Error bars display the standard error of the mean across three biological replicates. Student’s *t*-tests with statistically significant differences between mock and inoculated samples of the same time point were marked *p < 0.05 and **p < 0.01. The previously published data^27^ for wheat were included here for ease of comparison with *Brachypodium*.

As was observed in wheat^27^, genes encoding tryptophan metabolizing enzymes such as indole-3-glycerol phosphate synthase were up-regulated along with two genes putatively encoding aromatic-l-amino-acid decarboxylase (AADC) enzymes (Table 1). AADC enzymes are also up-regulated during Fusarium head blight infection in *Brachypodium*, resulting in increased accumulation of tryptamine and serotonin within infected plant tissue^23^. To examine whether tryptamine and serotonin were induced in *Brachypodium* seedlings during Fusarium crown rot as predicted, these compounds were quantified using LC-MS. Both tryptamine and serotonin were induced by *F*. *pseudograminearum* infection in *Brachypodium* at 3 and 7 dpi (Fig. 5). Interestingly, basal levels of serotonin appeared to be significantly higher in *Brachypodium* than wheat, as previously described for wheat as part of the same experiment^27^ and consistent with a previous study comparing metabolite accumulation in wheat and *Brachypodium* ^58^. The tryptophan derivative tryptamine plays a role in host resistance against fungal pathogens. In rice, this compound has been shown to impair both growth and virulence of *M*. *grisea* ^59^. Production of another tryptophan derivative, serotonin, is induced in wheat during infection by *Parastagonospora nodorum* ^60^, the causative agent of the glume blotch disease in wheat, and in *Brachypodium* spikelets during infection by *F*. *graminearum* ^23^.

### *Brachypodium* does not produce major wheat or barley associated indole-derived phytoalexins (benzoxazalinones and gramine) or secologanin

Within the RNA-seq dataset, three cytochrome p450 71c-like genes Bradi2g27782, Bradi3g36330 and Bradi3g36347 were induced by 6.4 fold, 2.5 fold and 1.9 fold, respectively. Two of these cytochrome encoding genes (Bradi3g36330 and Bradi3g36347) were induced at 7dpi (~9 fold and ~5 fold) and evidence for expression of the other annotated *CYP71c4* genes was observed. Close homologs of Bradi2g27782 were annotated as indole-2-monoxygenases in wheat (*Bx* genes), which function in the biosynthesis pathway for benzoxazolinones. Benzoxazalinone compounds have been previously identified widely across monocot species and also in a small number of dicot species^61^. The biosynthesis pathways for methoxy-6-benzoxazalin-2-one (MBOA) and 2-benzoxazalinone (BOA) have been well characterized in maize^62^ and rye^63^ respectively and bread wheat has been previously shown to produce both BOA and MBOA and genes encoding the first five enzymes catalyzing conversation of indole-3-glucosyl to BOA have been characterized in wheat^64^. The *Bx1* gene encodes an indole-3-glycerol phosphate lyase while *Bx2-5* encode cytochrome p450 monoxygenase enzymes designated cyp71c1 – cyp71c4. Interestingly, recent work has established *F*. *pseudograminearum* detoxifies benzoxazalinone compounds by utilizing a cluster of genes conserved in other *Fusarium* pathogens. *F*. *pseudograminearum* mutants with functional knockouts of these genes showed a high level of sensitivity to both BOA and MBOA as well as reduced virulence in infection assays^65,66^.

**Figure 6:**
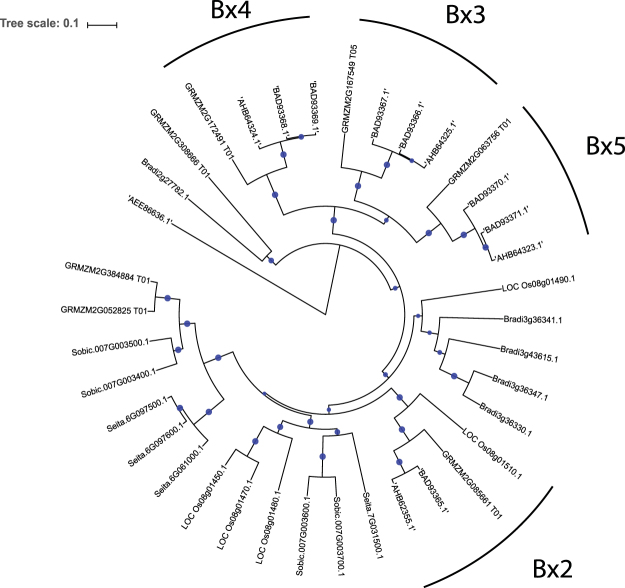
Phylogenetic comparison of putative *Bx* genes in *Brachypodium* with cytochrome p450 enzymes from rice, maize, sorghum, *Setaria italica* and wheat.

Genes annotated as encoding *CYP71c* enzymes in *Brachypodium* formed one gene cluster located on chromosome three, a pair of contiguous genes located more distally on chromosome three and five other genes located on chromosomes 1, 2 and 5. A phylogenetic analysis of putative *Bx* enzymes from *Brachypodium* alongside known wheat^67^ and maize^61^ *Bx* enzymes was performed. Results from this analysis (Fig. 6) indicated wheat and maize *Bx* genes clustered together in an ortholog specific manner while putative *Brachypodium Bx* genes grouped in a separate cluster. We then tested if *Brachypodium* can produce benzoxazalinones. Detection of BOA and MBOA within mock-inoculated and infected *Brachypodium* tissues was performed using LC-MS revealing *Brachypodium* does not produce either form of benzoxazalinone. In addition, we tested three *B*. *distachyon* ecotypes (Bd21-3, Koz-5 and Adi-16) and two *B*. *hybridum* ecotypes (BdTR4E and Bal-P4) for BOA and MBOA detection. These experiments showed that none of the genotypes tested produces BOA compounds, suggesting that the absence of benzoxazalinones is conserved across *Brachypodium* and may indicate a segmental loss during the course of evolution. In contrast, as we previously reported, wheat samples accumulate BOA and MBOA to high concentrations within both mock inoculated and infected samples^27^. Based on the degree of divergence observed between *Brachypodium* and wheat indole-3-glycerol phosphate lyase genes, it has been previously proposed that *Brachypodium* might not produce benzoxazolinones^68^. However, no metabolite analyses were available at the time to support this suggestion.

Interestingly, grasses produce benzoxazalinones or gramine but rarely both^69,70^. For instance, cultivated barley does not produce benzoxazalinones, instead producing gramine as an indole derived phytoalexin. Since *Brachypodium* shares a closer phylogenetic relationship with barley than wheat^13^ it seemed plausible that *Brachypodium* might produce gramine instead of BOA. We therefore tested *Brachypodium* for gramine production using LC-MS but no gramine was detectable (Fig. S4). Therefore, it appears that *Brachypodium* does not produce either of the major known phytoalexins from wheat or barley, supported by recent findings published during the preparation of this manuscript^70^.

Finally, we found a cluster of near contiguous genes occurring on chromosome *Brachypodium* chromosome 2 annotated as secologanin synthase genes which were up-regulated in a range from ~1.8-9.6 fold during *F*. *pseudograminearum* infection. Secologanin functions as a precursor for monoterpenoid and indole alkaloids^71^; compounds with anti-fungal activity^72^. Secologanin was not detected in *Brachypodium* tissue samples under mock or *F*. *pseudograminearum* infection though it was detected in wheat samples (Fig. S4).

## Conclusion

*Brachypodium* is susceptible to a diverse range of pathogens that infect cereals including *F*. *pseudograminearum* ^18^. The results presented in this study are consistent with a host responding to a biotic stress through transcriptional activation, leading to increased primary and secondary metabolism, cellular signaling and transport of molecules across cellular membranes. The overall similarity in transcriptional change during response to infection inferred at the homolog level; several molecular responses and metabolic pathways were found to respond similarly in both *Brachypodium* and wheat. In particular, induction of genes involved in phenylalanine and tryptophan biosynthesis leading to induction of phenylpropanoids, salicylic acid, tryptamine and serotonin was observed, further suggesting that these responses were largely conserved between *Brachypodium* and wheat. Metabolite analysis confirmed *Brachypodium* species did not produce benzoxazalinones or gramine; suggesting *Brachypodium* may produce a novel phytoalexin with a homologous and transcriptionally active pathway. Future work dissecting the basis of resistance to FCR will greatly benefit from the application of *Brachypodium* as a model system and will inform future wheat improvement strategies to increase resistance to FCR in wheat cultivars.

## Materials and Methods

### Pathogen Inoculations, Nucleic Acid Preparation and Sequencing

A laboratory based infection assay^73^ was performed using *Brachypodium* line Bd21-3^74^ to observe global transcriptional changes during infection by *F*. *pseudograminearum*. Seeds were germinated on Whatman^®^ filter paper placed in 150 mm × 25 mm Corning^®^ Petri dishes (Corning, catalog number: CLS430599). Seedlings (three days post-germination) were then carefully removed from the filter paper and placed into 50 mL Falcon^™^ tubes (VWR, catalog number: 14-432-22). Seedlings were then immersed in 3 mL of *F*. *pseudograminearum* spores (Isolate CS3427) suspended in sterile water and 0.01% Tween20 (1 × 10^6^ spores/mL) and incubated on a tube roller (Ratek Instruments, Boronia, Australia) for three minutes. The inoculum was then drained off and seedlings were carefully rolled into paper towel sheets so that shoot tissue was protruding from the top of the paper towel roll. Paper towel rolls were then placed inside 50 mL Falcon tubes and kept hydrated with addition of sterile water. Four biological replicates consisting of approximately 32 plants per replicate were conducted for both mock and *F*. *pseudograminearum* inoculated treatments. Leaf sheath enclosed tissue for each plant (both mock and *F*. *pseudograminearum* inoculated) was excised at three days post inoculation and immediately immersed in liquid nitrogen. Infection assays were performed concurrently with wheat infection assays described in Powell *et al*.^27^ so that transcriptome and metabolite analyses could be compared directly. Total RNA was extracted from homogenized tissue and quality control was performed using an Agilent 2100 Bioanalyzer system with all samples found to have adequate quantity and integrity (RIN > 9) for sequencing. As per Powell *et al*.^27^, validation of successful infection was performed through observation of severe symptom development in non-harvested plants at 14 dpi (Fig. S5) and significant differential expression of marker defense genes (*PR1*-like, *PR2*-like, *PR3*-like, *PR4*-like and *OPR-*like) within harvested plants (3 dpi) using qRT-PCR with the primers listed in Table S4. Messenger RNA (mRNA) was isolated using poly-A selection and 100 base pair (bp) paired-end libraries (non-stranded) were generated and bar-coded prior to sequencing. Sequencing was performed using an Illumina HiSeq. 2000 platform with all samples run on a single lane generating approximately ~45 gigabases across samples (read counts for individual libraries given in Table S5). Sequence files were deposited to the National Centre for Biotechnology Information (NCBI) Sequence Read Archive under BioProject ID PRJNA353032.

### Analysis of Differential Gene Expression using Tuxedo

Access to a high quality assembled and annotated genome (v2.1 annotation) for *B*. *distachyon* enabled use of the Tuxedo RNA-seq suite to perform differential expression analysis of the sequence data generated. While the capabilities and methodology has been thoroughly described and discussed elsewhere^75^, a brief description is provided here. In order to exclude sequencing errors where possible, sequence quality was analyzed using SolexaQA and paired-end reads were trimmed for PHRED score > 30 to read length 70 bp prior to alignment. Filtered and trimmed reads were aligned to the *B*. *distachyon* v2.1 genome annotation (Phytozome accessed 30/05/2014) using Tophat2 with Bowtie2 as the aligner. Alignment maps were assembled into transcript fragments and normalized to fragments per kilobase per million (FPKM) using Cufflinks, individual replicates for each treatment were concatenated using Cuffmerge and analysis of differential expression of genes was performed using CuffDiff to compare differential gene expression between mock and *F*. *pseudograminearum* inoculated samples. Statistical analysis was performed as part of the CuffDiff analysis to apply a false discovery rate and multiple comparison correction, (Bonferroni correction; adjusted *p* value < 0.05), enabling the calling of genes which are significantly differentially expressed between mock and infected conditions. For the identification of the alternative splicing events, cleaned reads trimmed to a length of 100 bp were retained and analyzed using the rMATs^33^, which takes into account a Bayesian and counts based model to identity the differential splicing events using junction based events mapping and junction based + reads on target mapping.

### Gene ontology assignment and enrichment testing

Gene annotations were inferred by BLAST2GO with enrichment testing performed as described in Conesa *et al*.^76^ using standard parameters (Fisher’s exact test). The global annotated collection (version 2.1) was used as a background reference for enrichment analysis with up-regulated genes ( > 2-fold) used as the test set. Enrichment of biosynthetic pathways was performed using KOBAS. For this analysis, global differentially expressed genes were used as BLAST queries against enzymes identified within KEGG pathways. A FDR correction (adjusted *p*-value < 0.05) was performed to identify statistically significant enrichment of pathways.

### Assessing Differential Transcript Expression using Real-time Polymerase Chain Reaction (qRT-PCR)

Separate crown tissue samples were harvested for gene expression analysis using qRT-PCR and for metabolite quantification. Additionally, leaf tissue from above the coleoptile was harvested for quantification of the defense hormones salicylic acid and jasmonic acid. A subset of genes of interest were selected for validation across a wider infection time-course incorporating earlier (1 dpi) and later (7 dpi) time-points in addition to 3 dpi to determine if differential expression remains consistent across repeated experiments (Fig. S1). Primers were designed to span intron-exon boundaries with preference to 3’ end junctions where possible. The gene encoding ubiquitin conjugating enzyme 18 (UBC18) was utilized as a reference gene as described in Hong *et al*.^77^ and Chambers *et al*.^78^.

### Identification of homologous genes between wheat and *Brachypodium*

Homologous genes within wheat and *Brachypodium* were identified using a reciprocal best BLAST hit (RBBH) approach (e-value < 1e^−5^). Global coding sequences from the International Wheat Genome Sequencing Consortium genome reference were retrieved (http://plants.ensembl.org/index.html on May 14^th^ 2014) and separated into subgenome specific groups. These sequences were independently used as BLAST queries against the *Brachypodium* global coding (*B*. *distachyon* annotation v2.1) sequence collection to identify direct homologs using an RBBH approach (e-value < 1e^−5^). This enabled identification of a single wheat homolog in one or more of the wheat subgenomes for the global complement of *Brachypodium* genes.

### Infection assay for assessing the role of toxin in disease

Development and validation of *F*. *pseudograminearum Tri5* mutants has been previously described^27^.

Pot-based inoculation assay: Pot-based disease assays were conducted in the CSIRO Agriculture Controlled Environment Facility (CEF) in Brisbane set to daytime temperature of 22 °C and 18 °C night with ambient light. Relative humidity was maintained at ~60%. *Brachypodium distachyon* (Bd21-3) seeds were stratified and vernalized at 4 °C for 14 days before placing them in the growth chamber to germinate. Seedlings (3–4 days post germination) were immersed within 5 mL of spore solution (10^6^ spores/mL) in 50 mL screw-top tubes on a tube roller for 3 min and then planted into 10 cm ANOVA pots containing Searles® potting mix (5 seedlings/pot). Four replicate pots were produced for each isolate tested as well as one mock-inoculated (no pathogen) replicate as a control. After planting, pots were watered in thoroughly and subsequently watered as required. Symptoms were scored using a qualitative scoring scale (0–5) at 7 and 14 days post inoculation.

Root rot pathology assay: Prior to sterilisation, the lemma was removed from *Brachypodium* seeds using forceps. Seeds were then surface sterilised using a 3% available hypochlorite solution for three minutes followed by 70% ethanol for three minutes and rinse 3–4 times with sterile water to remove residual hypochlorite and ethanol. 10–15 seeds were placed on two autoclaved Whatman™ No. 1 filter paper placed inside a 150 mm × 25 mm Corning^®^ Petri dish (Corning, catalog number: CLS430599). Filter papers were dampened with 8 mL of sterile distilled water. Plates were kept at 4 °C for one week to stratify the seeds before removing from the cold and transferring to the growth chamber under 16 hour light/8 hour dark at 22 °C. After *Brachypodium* seedlings had germinated and produced a primary root 3–4 cm in length, *Fusarium*-colonized agar plugs were placed mycelium side down on the root approximately 2 cm away from the seed. Under sterile conditions, the wide end of a sterile 200 µL pipette tip was used to excise round plugs from the growing edge of the plate. Plates were sealed with Parafilm or plate wrap and incubated in a growth cabinet under 16 hour light/8 hour dark at 22 °C. *Brachypodium* seedlings were observed for development of disease symptoms and scored for disease severity at 7 and 14 dpi.

### Quantitation of metabolites using LC-MS

Some of the coleoptile tissue from the infection assay which had been flash frozen was also used for metabolite analysis. The samples were ground to a fine powder using a Retsch ball mill and metabolites were extracted with 100% methanol overnight at room temperature before adding an equal volume of milliQ water and vortex mixing. Samples were then spun down using an Eppendorf 5424 benchtop micro-centrifuge (10,000 xg for ten minutes). The supernatant (500 µL) was aliquoted into clean tubes and centrifuged again to pellet protein and cellular debris with the supernatant used for analysis. Extracted plant samples were subjected to liquid chromatography multiple reaction monitoring mass spectrometry (LC-MRM-MS) as described in Powell *et al*.^27^ Five microliters of each sample was injected to a Shimadzu Nexera UHPLC. For detection of benzoxazalin-2-one, 6-methoxy-benzoxazalinone and secologanin, samples were passed through a Kinetex C18 1.7 µm column (Phenomenex 2.1 mm × 100 mm) with parameters given in Powell *et al*.^27^ while for detection of gramine, serotonin and tryptamine, samples were passed through a Kinetex HILIC 2.6 µm column (Phenomenex 2.1 mm × 100 mm) at 0.4 mL/min over 15 minutes at 40 °C with the following gradient: 100% solvent B for two minutes, a linear gradient from 100–60% solvent B over six minutes, a linear gradient from 60–5% over two minutes, followed by two minutes at 5% solvent B and an equilibration at 100% B. The mobile phase consisted of solvent A (0.1% formic acid/99.9% water) and solvent B (0.1% formic acid/90% acetonitrile/9.9% water). The mobile phase consisted of solvent A (0.1% formic acid/99.9% water) and solvent B (0.1% formic acid/90% acetonitrile/9.9% water). Mass spectrometer run parameters were applied as described in Powell *et al*.^27^. Each metabolite was detected by measuring four precursor-to-product ion (MRM) transitions and quantified by one transition (Table S6). Peaks were integrated using MultiQuant 3.0 (AB Sciex), the detection limit was set at a signal to noise ratio (S/N) of > 3 and peaks with a S/N > 7 were quantified. Standards were calculated from the average of two technical replicates and experimental samples were an average of two technical replicates and four biological replicates. Data was graphed and analyzed in Microsoft Excel. Standards for tryptamine, serotonin hydrochloride, benzoxazalin-2-one, 6-methoxy-benzoxazalinone, gramine and secologanin were obtained from Sigma (Sigma, MO, USA).

JA and SA were quantified using the method reported in Miyazaki *et al*.^79^ with the same sampling strategy described in Powell *et al*.^27^.

## Electronic supplementary material

Supplemental figures

Table_S1_Differentially_Expressed_Genes_Brachy

Table_S2_GO_term_enrichment_analysis

Table_S3_Alternative_splicing_events

Table_S4_qRT-PCR_primers

Table_S5_read_counts_for_sequence_files

Table_S6_MRM_transitions
