# Supplementary material for: Transcriptome analysis of Brachypodium during fungal pathogen infection reveals both shared and distinct defense responses with wheat
Source: Sci Rep. 2017 Dec 8;7:17212. doi: 10.1038/s41598-017-17454-3 (PMC5722949; doi:10.1038/s41598-017-17454-3)
Supplement: Supplementary file 1 — Supplemental figures [file 41598_2017_17454_MOESM1_ESM.pdf]

Transcriptome analysis of *Brachypodium* during fungal pathogen infection reveals both shared and distinct defense responses with wheat

Jonathan J. Powell[1,2*], Jason Carere[1], Gaurav Sablok[3], Timothy L. Fitzgerald[1], Jiri Stiller[1], Michelle L. Colgrave[1], Donald M. Gardiner[1], John M. Manners[4], John P. Vogel[5], Robert J. Henry[2], Kemal Kazan[1,2*]

[1]Commonwealth Scientific and Industrial Research Organization Agriculture and Food, St Lucia, Queensland 4067, Australia.
[2]Queensland Alliance for Agriculture and Food Innovation (QAAFI), University of Queensland, St Lucia, 4067, Queensland Australia;
[3]Plant Functional Biology and Climate Change Cluster (C3), University of Technology Sydney, PO Box 123, Broadway, NSW 2007, Sydney, Australia;
[4]Commonwealth Scientific and Industrial Research Organization Agriculture and Food, Black Mountain, Australian Capital Territory 2601, Australia;
[5]Joint Genome Institute, United States Department of Energy, Walnut Creek, CA, 94598, USA.

*Corresponding authors: J.J. Powell and K. Kazan;
E-mails: jonathan.powell@csiro.au and kemal.kazan@csiro.au;
Telephone: +61 7-3214-2340 and +61 7-3214-2678.

Figure S1: Heatmap showing fold-change for the most differentially expressed genes (top 100).

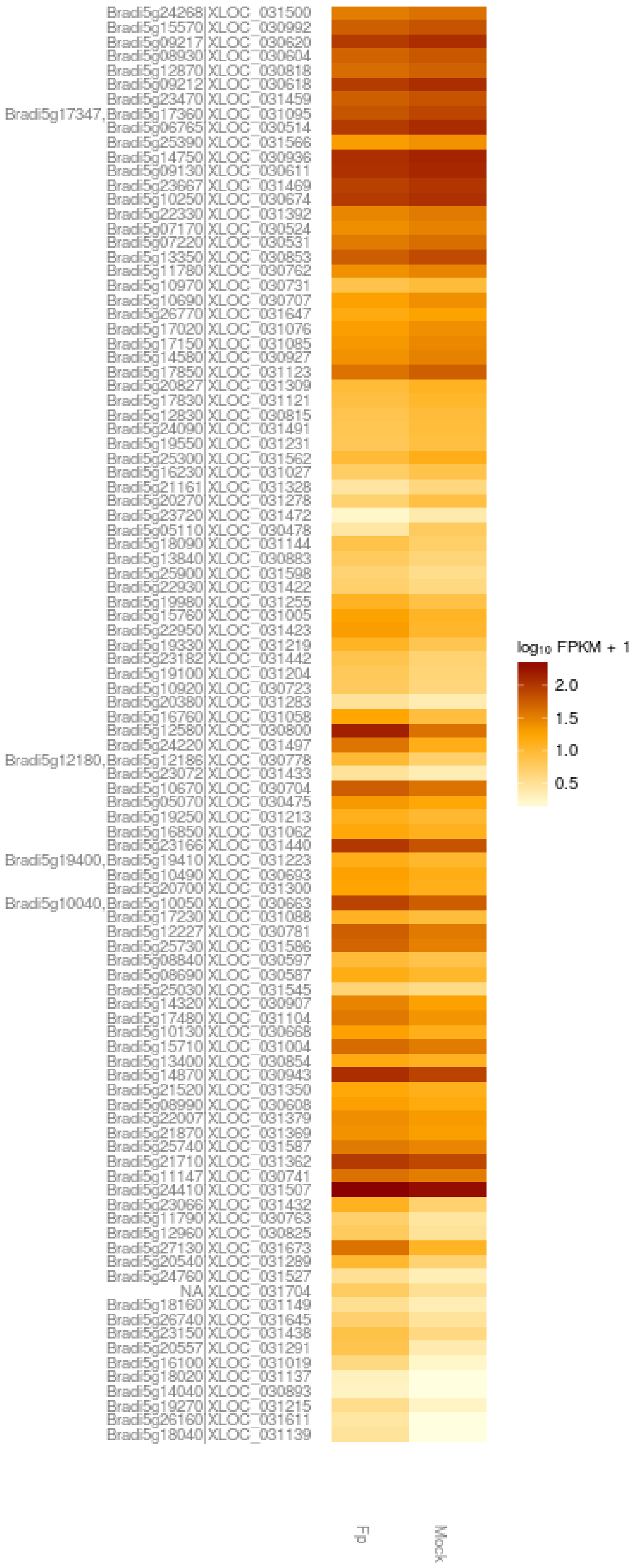

Figure S2: Relative expression of *F. pseudograminearum* responsive genes across an infection time-course using qRT-PCR. *Fp* : *F. pseudograminearum;* dpi: days post-inoculation.

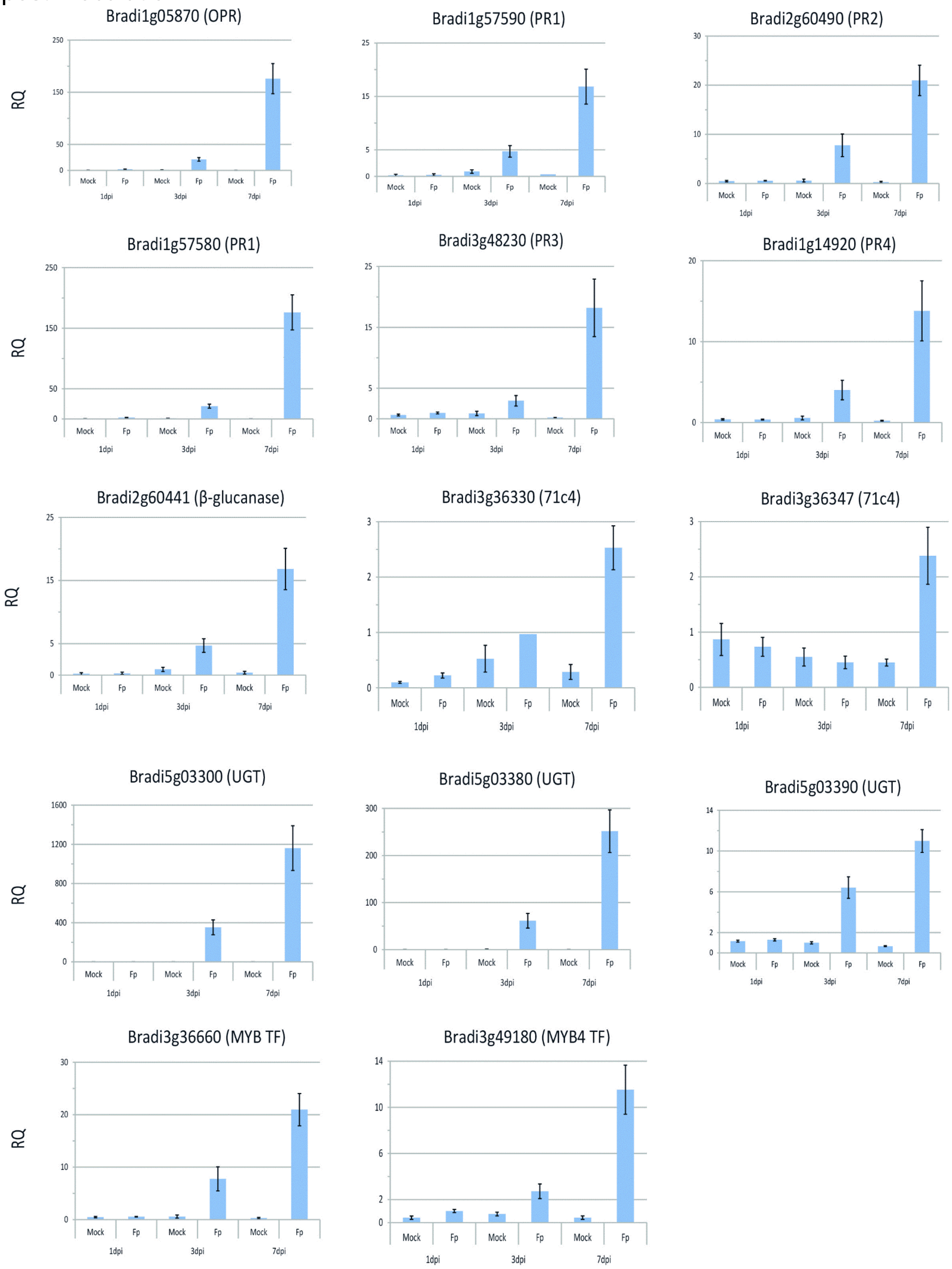

Figure S3: Acyclic map showing enriched GO terms in hierarchical arrangement.

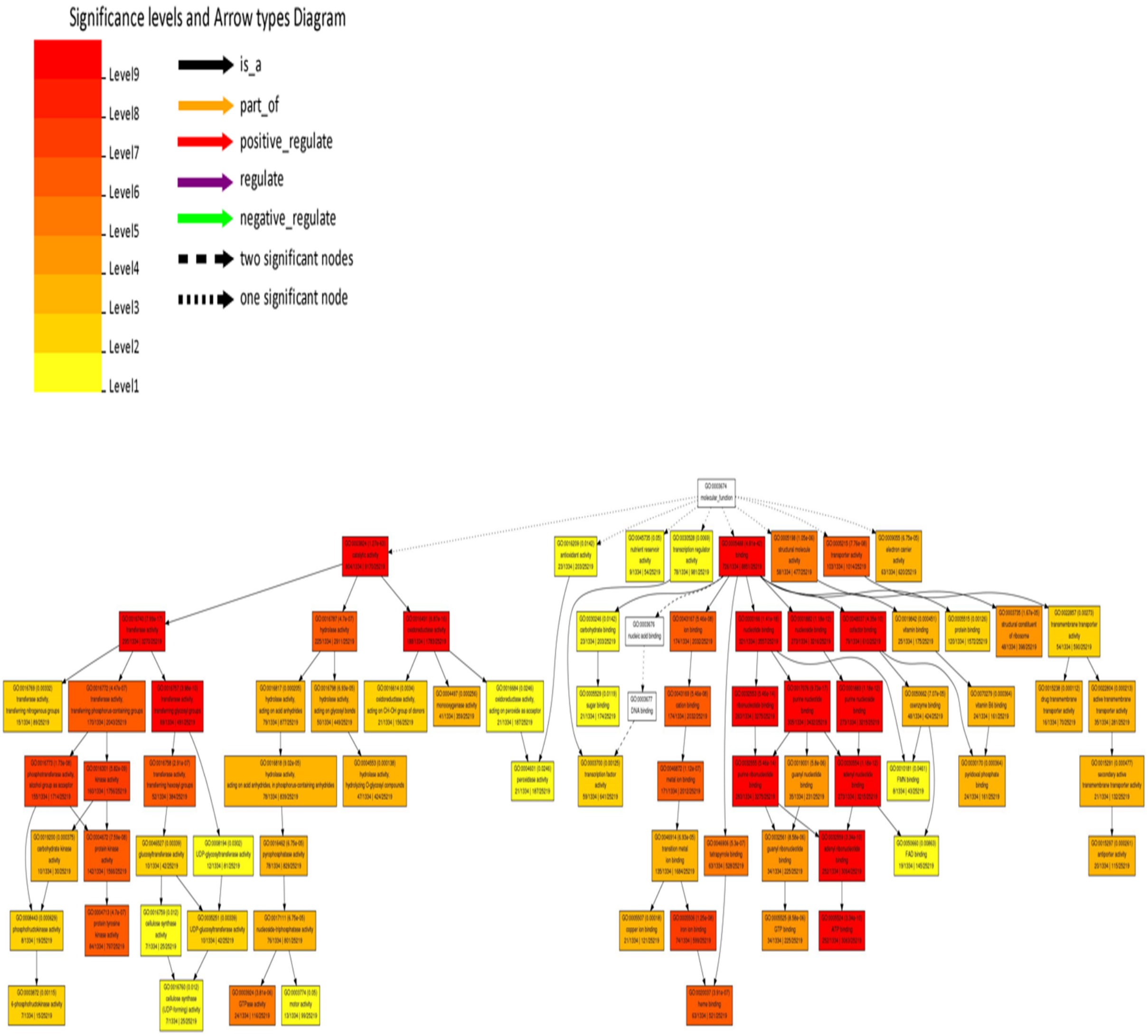

Figure S4: Chromatograms for gramine (a.), BOA (b.), secologanin (c.), and MBOA (d.) with metabolite standards (top graph) compared to *Brachypodium* samples (bottom graph).

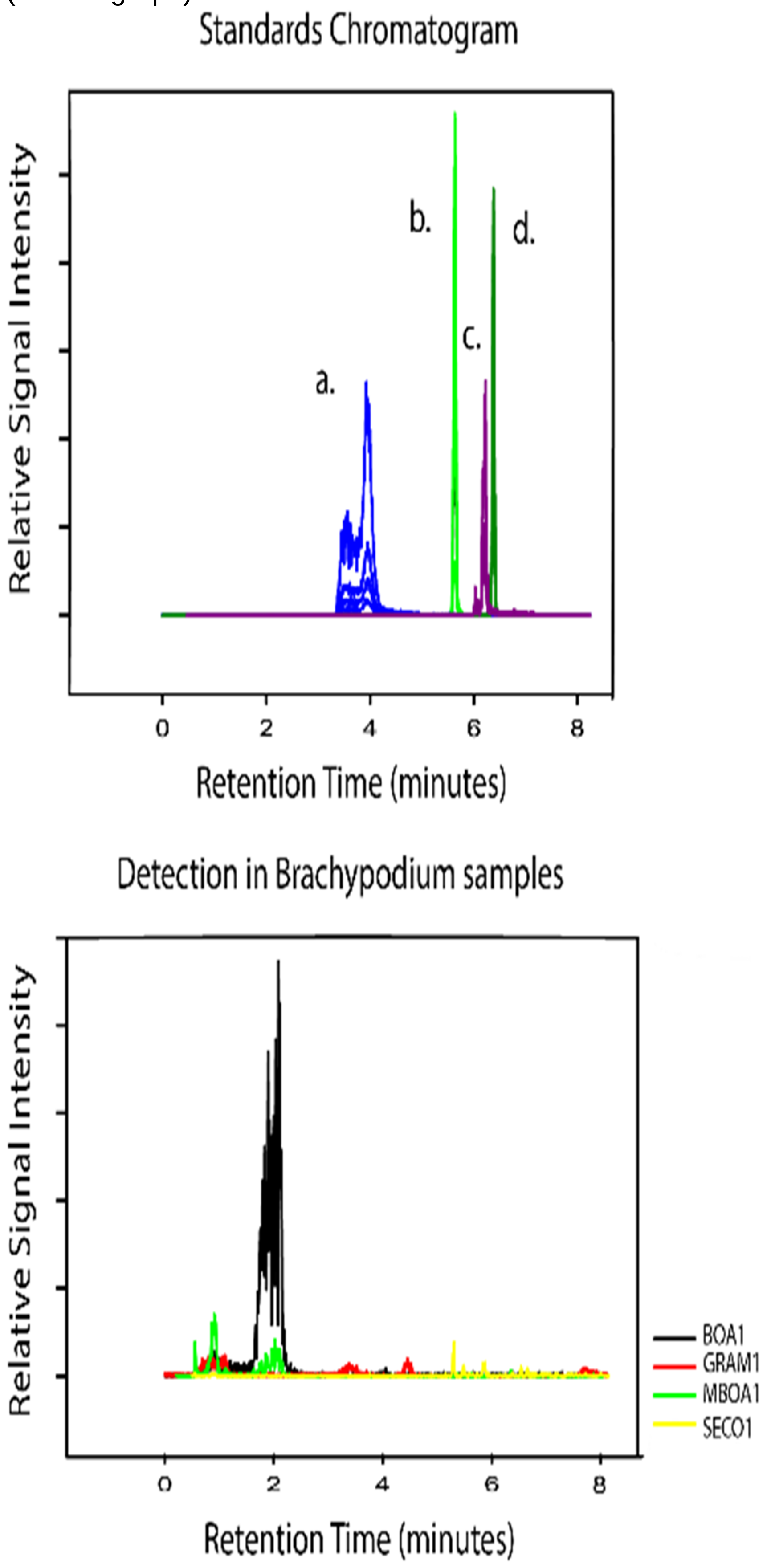

Figure S5: Representative photographs of disease symptoms on *Brachypodium* seedlings at 14 days post inoculation.

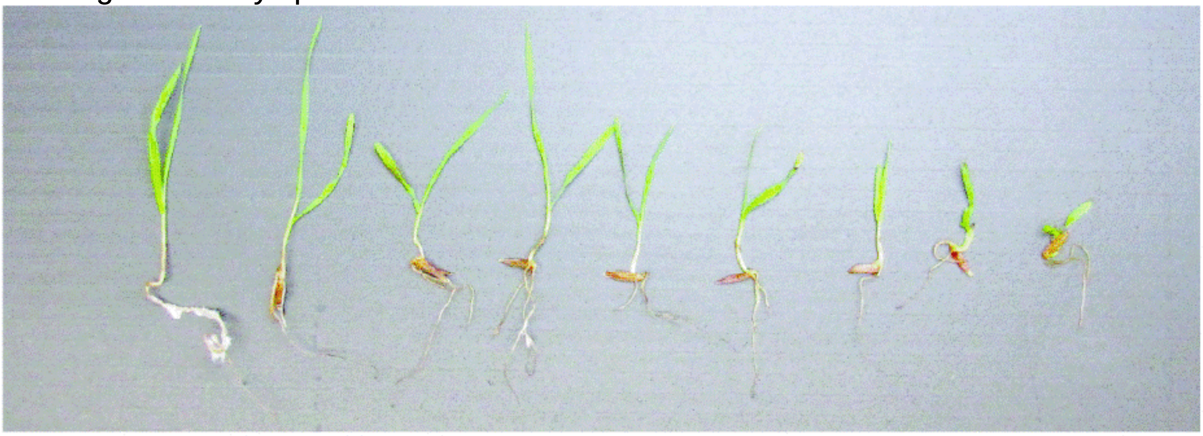

mock inoculated

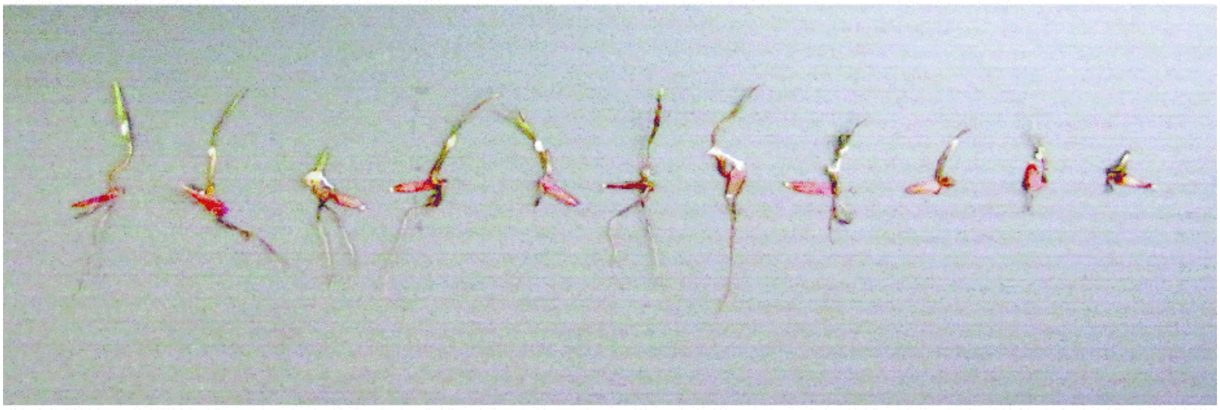

F. pseudograminearum
inoculated
